# Assisting Screening of Adolescent Depression With Large Language Models as Symptom Detectors: Algorithm Development and Validation

**DOI:** 10.2196/71614

**Published:** 2026-10-21

**Authors:** Mariia Ignashina, Paulina Bondaronek, Parand Shams, Joanne Osuchukwu, Daniel Santel, John Pestian, Julia Ive

**Affiliations:** 1 Queen Mary University of London London United Kingdom; 2 University College London London United Kingdom; 3 Division of Biomedical Informatics Cincinnati Children's Hospital Medical Center Cincinnati, OH United States; 4 Department of Biostatistics Health Informatics and Data Sciences University of Cincinnati, College of Medicine Cincinnati, OH United States

**Keywords:** mental health clinical notes, adolescent depression, large language models, zero- and few-shot settings, semiautomated depression screening

## Abstract

**Background:**

Depression prevalence is rising in adolescents and young adults (ages 10-24 years). Traditional screening methods for depression, such as the Patient Health Questionnaire (PHQ-9), are particularly challenging for adolescents. AI has potential to help with this, but the scarcity of annotated datasets highlights the need for off-the-shelf approaches. In this study, we investigate the feasibility of using state-of-the-art large language models (LLMs) to detect depressive symptoms in adolescent settings (ages 15-17 years).

**Objective:**

This study aimed to evaluate the feasibility of using in-context learning with LLMs to identify depressive symptoms in free-text clinical notes for adolescent populations, to evaluate the feasibility of off-the-shelf LLM configurations in detecting PHQ-9 symptom-related information under zero- and few-shot settings, and to demonstrate the potential clinical utility of LLM-driven evidence extraction through an interpretable AI-based depression screening tool.

**Methods:**

We examined free text of the electronic health records (EHRs) of adolescent patients with the diagnosis of depression or related mood disorders (age groups 15-17 years, 924 patients) from the Cincinnati Children’s Hospital Medical Center. We leveraged the combination of the Beck Depression Inventory (BDI) and the PHQ-9 to develop tailored categories suited for adolescent depression symptoms. We applied 3 state-of-the-art LLMs (Llama, smaller FLAN-T5, and Phi) to identify these symptom categories in zero- and few-shot configurations, allowing us to assess their performance without additional task-specific training. A word-search model was used as the baseline comparator.

**Results:**

We manually annotated clinical notes for 22 patients across 16 depression-related symptom categories. Our analysis shows that all LLMs substantially outperformed the word-matching baseline. Within the evaluated prompting configurations, FLAN-T5 achieved the highest precision (average *F*_1_-score=0.65, precision=0.78) and strong performance in detecting rare symptoms such as sleep problems (*F*_1_-score=0.92) and self-loathing (*F*_1_-score=0.80). Phi offered a balanced performance (precision=0.44, recall=0.60), while Llama 3 achieved the highest recall (0.90) but tended to overgeneralize, making it less suitable for precise screening. Within this pilot study, the best-performing LLM exceeded the baseline *F*_1_-score by 0.43. Finally, we demonstrate the clinical utility of FLAN-T5 annotations as features in a machine learning model, which distinguished depression cases from controls with high precision (0.78).

**Conclusions:**

We used a range of popular LLMs to identify indicators of adolescent (ages 15-17 years) depression symptoms in mental health notes. Within the evaluated configurations, FLAN-T5 demonstrated the most favorable balance between precision and recall. These results support the feasibility of using off-the-shelf LLMs for symptom detection in adolescent clinical notes, while future work using standardized prompting strategies and task formulations will help establish more definitive comparisons between model architectures. The computational efficiency of FLAN-T5 further supports its potential for deployment in resource-limited clinical settings. Our findings require further validation for broader populations and other mental health conditions.

## Introduction

### Background

Depression is a complex mental health condition that affects 4.7% [1] across various age groups. Population-based studies suggest that the point prevalence of depressive symptoms among children and adolescents in the United States ranges from 10% to 15% [2]. For example, the Centers for Disease Control and Prevention (CDC) estimates that approximately 8% of adolescents experience major depressive disorder [3]. It is typically characterized by persistent sadness, loss of interest or pleasure (anhedonia), and a broad range of emotional, cognitive, and physical symptoms. Unlike temporary emotional fluctuations, depressive disorders are marked by their severity, functional impairment, and resistance to relief from everyday positive experiences or social interactions [4].

Young people (ages 10-24 years) are particularly vulnerable to depression, as it coincides with critical stages of social, emotional, and cognitive development. In recent years, there has been a sharp rise in depression within this age group, especially among females [3]. Many young individuals also exhibit “subthreshold depression” [5], where depressive symptoms are present but do not meet the full diagnostic criteria for major depressive disorder.

A commonly used tool for assessing depression is the Patient Health Questionnaire (PHQ-9). The PHQ-9, developed by Kroenke et al [6], is a validated screening instrument based on the *Diagnostic and Statistical Manual of Mental Disorders*, Fourth Edition (DSM-IV) criteria for major depressive disorder. It includes 9 questions in which individuals rate the frequency of depressive symptoms over the past 2 weeks on a scale of 0 to 3. The total score indicates the severity of depression, with higher scores reflecting greater impairment.

However, screening is difficult to implement in the pediatric and adolescent primary care setting, with traditional approaches that rely on multiple informants often producing discrepant findings [7]. Beyond this, clinicians in the primary care setting vary in how they consider risk factors and interpret symptoms [8], and clinicians may struggle to disentangle overlapping symptoms [9].

Most research on detecting depression using machine learning focuses on analyzing social media data [10], where challenges like metaphorical language, sarcasm, and data scarcity make the task particularly complex. The inherent data sparsity problem has led to semiautomatic approaches to data collection via identifying self-expressions of mental illness diagnoses [11].

Early studies, such as Yazdavar et al [12], address the depression detection problem by leveraging vocabulary matches and traditional models like latent Dirichlet allocation (LDA) to identify depression-related topics based on PHQ-9 criteria. Building on this foundation, Yadav et al [13] explore the complexities of figurative language, such as metaphors and sarcasm, in depressive social media posts using fine-tuned transformer-based BERT (Bidirectional Encoder Representations from Transformers) models. More recent studies such as Mendes et al [14] emphasize that the detection of subtle, figurative expressions of depression remains a significant hurdle even for the powerful pretrained models.

Large language models (LLMs) have demonstrated significant potential in mental health care due to their advanced linguistic capabilities and generalization ability [15]. A key strength of LLMs is their capacity for off-the-shelf analysis, where they can process and extract insights without requiring annotated training data. This capability is especially valuable in mental health, where annotated datasets are limited due to ethical and privacy concerns. In health care applications, smaller models like FLAN-T5 (Finetuned Language Net, Text-to-Text Transfer Transformer) have shown promising results in off-the-shelf evidence detection, identifying cases such as postpartum hemorrhage more effectively than traditional methods [16]. Similarly, in social science research, FLAN-T5 has outperformed human crowdworkers in producing high-quality free-form explanations, highlighting its potential for off-the-shelf data annotation [17].

Beyond information detection, LLMs are being explored for diverse applications in mental health, particularly where they can reduce clinician workload. For example, models have been used to code qualitative patient interview transcripts, achieving moderate agreement with human annotators (Cohen κ=0.4) and successfully identifying both common and unique concerns [18]. In another example, language model-based tools have supported therapy delivery by automating session recording and analysis [19]. Clinicians reported reduced cognitive burden and improved ability to focus on patients, while both clinicians and patients expressed high satisfaction.

Despite these promising capabilities, significant challenges remain when using LLMs directly in mental health contexts. Key concerns include hallucinated content, biased outputs, and random guesses when models attempt to classify data without adequate context [20]. Recent evidence shows that LLMs will confidently elaborate on fake or impossible medical conditions, laboratory values, or physical signs in up to 82% of test cases [21]. Additionally, LLMs have been shown to perpetuate harmful, race-based medical misconceptions [22]. These issues undermine their reliability as standalone diagnostic tools, even when human oversight is involved. To address these limitations, a safer approach is to leverage LLMs for evidence detection rather than classification. By focusing on detecting text-based insights for clinical interpretation, this method reduces the risks associated with hallucinations and inaccuracies while preserving the benefits of automated data processing.

In general domains, LLMs have proven effective for retrieving supporting evidence in question-answering tasks [23]. These models are good at finding important information in large amounts of text, which professionals can then review to help with clinical decisions. Using LLMs as smart search tools is a practical and low-risk option for health care.

Recent research has extended these capabilities to mental health, using LLMs to annotate symptoms of conditions such as depression from online posts and psychological interviews [24-27]. These symptom annotations provide valuable inputs for downstream analytical models, improving the robustness of mental health screening.

LLMs can play a crucial role in improving mental health screening, particularly where data are limited and depressive symptoms vary widely. Their advanced language processing capabilities enable them to efficiently extract and interpret relevant information from unstructured text, making them well-suited for this complex task.

### Study Aim and Objectives

This study aimed to evaluate whether advanced LLMs can help detect depressive symptoms in adolescent primary care by identifying relevant text segments in electronic health records (EHRs), using PHQ-9 criteria.

Rather than relying on LLMs for diagnostic decisions, the models are used to detect potentially relevant text segments, ensuring that clinicians maintain full control over interpretation and decision-making. This approach adheres to ethical principles by preserving human judgment in the diagnostic process, offering a supportive tool for mental health screening in the challenging adolescent setting (ages 15-17 years).

The main objectives of this study were to (1) assess the feasibility of using in-context learning with LLMs in identifying depressive symptoms in free-text clinical notes for adolescent populations; (2) evaluate the feasibility of the performance of three leading LLMs in detecting PHQ-9 symptom-related information, using manually labeled EHRs as the reference standard; (3) demonstrate the potential clinical value of LLM-driven evidence detection to improve mental health screening and reduce data sparsity issues using an example of an interpretable AI-based depression screening tool.

## Methods

### Ethical Considerations

This study was approved by the Institutional Review Board of Cincinnati Children’s Hospital (STUDY2020-0942).

This was a retrospective study conducted with locally installed models on institutional servers. No human participants were enrolled, and no remuneration was provided. To protect privacy and in line with institutional policy, we do not publicly share source code or any patient-level data. The manuscript contains no information pertaining to actual patients.

### Study Design

This study involved a retrospective cohort analysis of pediatric EHRs collected from the Cincinnati Children's Hospital Medical Center (CCHMC) over an 11-year period.

### Data Collection

#### Overview

We used the CCHMC foundational database, which contains EHR data from approximately 1.3 million unique pediatric patients treated between January 1, 2009, and March 31, 2022, encompassing over 63 million clinical notes.

For this study, we analyzed clinical notes recorded between January 2011 and December 2021. PHQ-9 scores were primarily sourced from observation tables, standardized charts used to record patient parameters and observations. To extract scores from free-text notes, we searched for 3 verified phrases: “PHQ-9 Score:”, “PHQ-9 Total Score =”, and “PHQ9 score:”. Instances preceded by terms such as “Last” or “Most Recent,” where the PHQ entry date did not match the clinical record, were excluded to prevent duplication. Overall, only a negligible proportion of PHQ-9 scores were obtained from text, as most were captured in structured tables.

#### Inclusion Criteria

Patients who had a depression diagnosis with at least one visit recorded in the EHR during the 18 months prior to diagnosis. Diagnoses were identified using ICD-10 (*International Statistical Classification of Diseases and Related Health Problems*, Tenth Revision) codes corresponding to depressive disorders and related mood disorders, including major depressive disorder and its subtypes (F32.0, F32.1, F32.2, F32.3, F32.4, F32.5, F32.81, F32.89, F32.9, F33.0, F33.1, F33.2, F33.3, F33.40, F33.41, F33.42, F33.8, F33.9, or these ICD-9 [*International Statistical Classification of Diseases and Related Health Problems*, Ninth Revision] codes: 296.2, 296.21, 296.22, 296.23, 296.24, 296.25, 296.26, 296.3, 296.31, 296.32, 296.34, 296.33, 296.35, 296.36). All clinical notes attached to visits prior to the diagnosis date were included for analysis.

#### Exclusion Criteria

Patients with incomplete visit records or missing key clinical data were excluded from the analysis.

### Symptom Annotation Process

The annotation process was primarily guided by the 21 questions from the Beck Depression Inventory (BDI) [28], which were further augmented with additional symptoms from PHQ-9. Domain experts considered the BDI’s comprehensive scope better suited for identifying depression in adolescent patients. Additionally, PHQ-9’s semantics posed interpretability challenges for LLMs, reinforcing the need to use BDI as the primary framework.

After a clinical domain expert with experience in the adolescent age group helped the authors develop the list of relevant symptom manifestations, 2 annotators independently extracted symptom mentions from 15 notes. All disagreements were resolved through discussion until full consensus was reached. Following this, one of the annotators proceeded with the remaining work.

To further assess annotation reliability, we conducted an additional validation on a random sample of 30 notes independently reviewed by 2 annotators (PS and JO). Approximately 35% of each note contained annotatable content (~300 sentences in total), with only 10 disagreements (3.3%), corresponding to an interrater agreement of 96.7%. These discrepancies were limited to a small number of borderline cases, such as whether weight-related statements should be labeled as “weight change” versus “low appetite,” differences in how much contextual text (eg, regarding household members) should be included within an annotation span, and whether mentions referred to current versus past symptoms.

A total of 85 clinical notes from CCHMC were manually annotated. These notes were sourced from departments including adolescent medicine, endocrinology, primary care, pediatrics, and gastroenterology, covering 22 randomly selected patients (13 females and 9 males) aged 15-17 years (from a larger pediatric database spanning ages 6-24 years). The average note length was 425 words, and the dataset maintained a balanced class distribution, with 50% of the notes belonging to a patient with at least one positive PHQ-9 answer in recent time.

Annotations were performed at the sentence level, explicitly marking the presence or absence of depressive symptoms. This resulted in binary labels (positive or negative) for each symptom in the note. Thus, if a note contained any sentence indicating “irritability,” the entire note was labeled positive for that symptom. For example, a hypothetical note containing “The patient called regarding their Emergency Room visit yesterday. They stated that even though they refused to answer any of the clinicians’ questions last night, they have since calmed down and would like to come to the clinic again” was labeled positive for “irritability.”

We made efforts not to conflate historical data with the current patient state. Many notes contained information about previous patient complaints, which were almost always explicitly dated. In such cases, if the symptom occurred more than a month prior to the EHR’s dating, we labeled the symptom as *historical* and did not flag the note for having it. This was particularly important because many patients had a continuous history of mental health concerns, and our goal was that the system helps track their progress instead of repeatedly reporting outdated issues.

We carefully handled conflicting data. When patients or their caregivers denied experiencing certain symptoms, we labeled their statements as negative evidence and did not label the note based on them. In cases where only the caregivers mentioned a symptom or there were disagreements between the parties on the symptom’s presence, we marked the symptom as present. Hence, if the caregiver reported that the patient was undereating, we would mark the note positive for low appetite regardless of the patient’s self-report. This decision was motivated by trying not to miss any potential risk indicators. To prioritize observable behavior over self-reported statements, if a patient’s behavior contradicted their self-report, we marked symptoms as present. For example, if the patient had missed several weeks of school, but claimed that they did not have trouble going to school, we still marked their note positive for not going to school.

### Challenges in Annotation

Several challenges were encountered during the annotation process: (1) The descriptions of symptoms varied widely across patients, making consistent annotation difficult. For instance, while some notes explicitly mentioned concrete symptoms such as “loss of appetite,” others used ambiguous terms like “weight concerns” without specifying whether the change was related to increased or decreased appetite. (2) Adolescent patients often struggled with self-awareness and articulation of certain symptoms, particularly those requiring introspection, such as guilt or self-loathing. These symptoms were less frequently documented, as they require direct elicitation from clinicians during consultations. (3) Notes written by different clinicians showed diverse styles and depth of reporting. For example, nurse notes frequently captured nuanced behaviors and subtle symptoms that might not have been documented in more formal physician notes. Behavior-based symptoms, such as “neglecting activities,” were described in varying contexts, such as “the patient quit drama club” or “the guardian needs to encourage the patient to do drama.”

In contrast to social media texts, where somatic signs are usually absent [14], our clinical data explicitly mention patients’ general physical states and behaviors. This allowed us to capture a broader range of symptoms, including physical manifestations, which are often overlooked in digital text analysis.

### Natural Language Processing (NLP) Models

Following previous research [16,17], we used the FLAN-T5 (a smaller language model, google/flan-t5-small) [29] in its default configuration. We also used the quantized Llama 3-70B (neuralmagic/Meta-Llama-3-70B-Instruct-quantized.w8a16) [30] and Phi 3.5 Mini (another compact model, microsoft/Phi-3.5-mini-instruct) [31] in their default configurations to detect symptom mentions in text. Considering the constraints of handling private clinical data and limited computational resources, we selected these models for their ability to be deployed locally, minimizing external exposure.

Due to memory constraints in the Llama and Phi models, we limited input size to 6000 characters (approximately 1500 tokens, the maximum we could accommodate). This led to truncation in about 18% (15/85) of notes. To reduce information loss, we retained the first 3000 characters and the last 3000 characters, omitting the middle section. This approach reflects the typical structure of lengthy clinical notes: patient concerns and contextual details are usually presented at the beginning, while the end contains a concise summary that reiterates key findings. The middle section often consists of detailed physical exam blocks and structured test results, which are less critical for our task. In contrast, Llama 3 and Phi receive a larger context that includes summary sections which largely repeat earlier content, giving them an informational advantage. For FLAN-T5, we set the maximum input length to 512 tokens, corresponding to approximately the 75th percentile of note lengths in our dataset. For each model, we asked whether each text contained evidence of specific symptoms (binary classification). We describe the prompts we used for each model in Textboxes 1 and 2.

**Sample few-shot Llama 3 and Phi prompt for “not going to school” (real text was paraphrased).**

**System:** You are a medical AI assistant.

**User:** Here is an EHR note: “Chez Dias is a 15 y.o. female Latina adolescent.

Chief Complaints: SoB, Overeating, Acid Reflux.”

Does it contain evidence that the patient is not going to school?

**Assistant:** No.

**User:** Here is an EHR note: “Jeff has not been able to go out of house due to his fear of crashing during an episode.

He had to be home-schooled this year.”

Does it contain evidence that the patient is not going to school?

**Assistant:** Yes: “He had to be home-schooled this year.”

**User:** Here is an EHR note: “Does your child skip school?

Are they running around town? Do you think they are using drugs? Call the Hospital to find out the best ways of taking care of your teenager or visit our website.”

Does it contain evidence that the patient is not going to school?

**Assistant:** No.

**User:** Here is an EHR note: *note text*. Does it contain evidence that the patient is not going to school?

**Assistant:**

**Sample FLAN-T5 prompt for “no motivation” symptom (real text was paraphrased).**

**Premise:** This is an EHR note: *note text*.

**Hypothesis:** The patient is not taking proper care of themselves and their health.

Does the premise entail the hypothesis?

According to best practices in the domain [32,33], we used few-shot prompting for Phi and Llama, using 3 in-context examples. These examples were designed based on insights gained from observing the data. For FLAN-T5 prompts, we did not use few-shot examples, again adhering to standard practices.

As a baseline, we implemented a rigid word-level mapping approach using key parts of the questions. For instance, for the question “Does it contain evidence that the patient is not going to school?” we matched using the words “going” and “school.” This baseline is intended as a naive lower bound rather than a competitive clinical NLP approach. The simple word-search baseline did not account for clinical negation or contextual modifiers, which could improve its accuracy. This limitation may provide an advantage to LLM-based models, which are capable of interpreting context and handling negation.

### Utility of LLM Detection for Depression Screening

Our LLM annotations aim to provide meaningful insights that could potentially aid in clinical screening. However, directly evaluating their utility with real-life doctors is challenging due to the complexity and variability of clinical workflows. As a practical proxy for human judgment, we assess the usefulness of these annotations by using them as features in a range of traditional machine learning algorithms designed for clinical decision-making. This approach allows us to evaluate whether the annotations meaningfully contribute to distinguishing between case and control patients, which is an indirect measure of their potential impact in real-world clinical settings.

For this experiment, we focused on the 15-17-year-old cohort (see Multimedia Appendix 1). We first extracted all patients in this age group who had completed a PHQ-9 questionnaire, then identified those we could successfully match to controls, resulting in 462 case patients. Overall, we retrieved all available clinical notes recorded within 2 weeks of PHQ-9 completion for each patient, resulting in approximately 3000 notes across the entire case cohort. Controls were matched by age and gender, requiring birth dates within 30 days of the corresponding case patient. Additionally, controls had no history of depression diagnosis up to the case patient’s diagnosis date and had a clinical visit within the same 18-month window. This process yielded a final dataset of 924 patients (50% cases, 50% controls).

Building on our annotation experiment, we used FLAN-T5 to generate a symptom vector for each patient. This vector consisted of 16 dimensions (one dimension per question). Each dimension indicated the proportion of clinical notes of a patient containing specific symptoms as detected by FLAN-T5. These symptom vectors served as features for a range of traditional classifiers, including random forest, logistic regression, support vector machine (SVM), multilayer perceptron (MLP), and decision tree models. The goal was to determine whether the features derived by symptom detection could be distinctive enough to separate case and control patients.

The 85 manually annotated notes were included in this 924-patient cohort used for the downstream task. However, FLAN-T5 was used zero-shot, without task-specific fine-tuning, and the prompts were not optimized using these 85 notes. We hence consider the risk of data leakage to be minimal.

Classifier performance was evaluated using repeated stratified 5-fold crossvalidation with 20 repetitions (100 train-test splits), with 95% CIs derived from performance scores across repetitions and folds. Logistic regression was selected as the reference baseline given its widespread use. Differences in the area under the receiver operating characteristic curve (AUC-ROC), *F*_1_-score, precision, and recall relative to logistic regression were assessed using paired nonparametric bootstrap resampling of the out-of-fold predictions (2000 bootstrap samples). A difference was considered statistically significant if the 95% CI of the paired bootstrap difference did not include zero.

As a semantic baseline, we used the BigBird transformer model [34] to classify the most recent clinical note for each patient without any intermediate feature extraction. The model used a maximum input length of 512 tokens, which corresponds to approximately the 75th percentile of note lengths in our dataset, meaning that the most recent note was usually processed without truncation. Preliminary experiments using merged notes or longer clinical histories did not improve performance; therefore, the final reported configuration used only the most recent note, as it captured the most relevant clinical signal for this task. Both the BigBird baseline and the traditional machine learning classifiers were evaluated using the same 5-fold cross-validation procedure, allowing predictions to be generated for the entire dataset while ensuring that each fold was evaluated on held-out data.

## Results

### Cohort Characteristics

The cohort included 28,172 pediatric and adolescent patients, aged between 6 and 24 years; the percentages below refer to the 24,890 patients in age groups with sufficient PHQ data (Multimedia Appendix 1). The smallest representation was in the 24-year-old group, while the largest was in the 15-year-old group. PHQ-9 completion was more prevalent among older patients, particularly those aged 16 years and above, with an average completion rate of 57% (14,121/24,890) across all age groups (at least one full PHQ-9 questionnaire completed). Detailed PHQ information beyond the binary indicator (“PHQ taken”) was missing for approximately 2% (520/24,890) of patients. See detailed statistics, including age-specific PHQ completion rates in Multimedia Appendix 1.

On average, approximately equal proportions of patients (13,997/24,890, 56%) completed at least one PHQ-9 or one PHQ-2, with PHQ-9 completion becoming more prevalent in older age groups. Among patients aged 16 years and above, 71% (7612/10,670) completed at least one PHQ-9, compared to 48% (5080/10,670) who completed at least one PHQ-2.

However, completion rates dropped noticeably for repeat PHQs: pooled across age bins, the proportion of patients completing at least one PHQ fell by 54 percentage points, from 98% (24,370/24,890) to 44% (11,006/24,890) completing two or more. Pooled across age bins, the proportion of patients completing at least one PHQ-2 fell by 40 percentage points, from 56% (13,872/24,890) to 15% (3,784/24,890) completing at least two PHQ-2s. The largest drop was at age 6, from 91% (86/94) to 22% (21/94), or 69 points. A similar decline was observed for full PHQ-9 completions, which fell by 34 percentage points, from 57% (14,121/24,890) to 23% (5,732/24,890). This drop reached 68 points at age 23, where none of the 68% (73/107) of patients with one PHQ-9 completed a second (0%, 0/107). This underscores the difficulty of obtaining repeated full diagnostic data. Percentages reported in the text are pooled proportions, calculated as the total number of patients meeting each criterion divided by the total number of patients across all age bins (N = 24,890). The ‘Av.’ row in Multimedia Appendix 1 instead reports the unweighted arithmetic mean of the age-specific percentages. This drop highlights the challenges clinicians face in gathering comprehensive depression-related data.

### Factors Contributing to PHQ-9 Data Sparsity

Several factors contributed to the observed data sparsity. PHQ-9 tests were commonly administered only in specific departments, such as primary care, limiting their broader use. Additionally, phone call scripts often provided supplementary insights into the onset of depression that were not captured through PHQ-9 questionnaires. Some patients admitted after suicide attempts scored relatively low on the item “Thoughts that you would be better off dead, or of hurting yourself in some way?” This likely reflects the time-specific phrasing of the response options: they may not have had such thoughts nearly every day or half the days in the past 2 weeks, but persistently for a very short period of time.

Certain symptoms assessed by the PHQ-9, such as being fidgety or restless, were particularly difficult for individuals to self-evaluate. External motivations, such as fear of stigmatization, could lead to inaccurate responses. In some instances, patients left the hospital before completing all required procedures. Furthermore, the PHQ-2 screening protocol dictated that patients testing negative for depression symptoms in the first 2 questions did not proceed to the remaining 7 questions of the PHQ-9, further contributing to the incomplete data.

### Symptom Annotation Results

We identified the following symptom categories from the clinical notes (all examples are paraphrased):

1. Not going to school:
  Interruptions in on-site schooling, for example, “The patient hasn’t been to school this month.” Related to BDI items 4 (“I am dissatisfied or bored with everything.”) and 15 (“I can’t do any work at all.”).
2. Neglecting activities:
  Interruptions in exercising or extracurricular activities, or absence of physical activities, for example, “The patient didn’t enrol into any after-school activities or clubs this year.” Related to BDI items 4 and 15.
3. No motivation:
  Lack of motivation in completing day-to-day tasks, such as brushing teeth, for example, “The patient has stopped following their diet plan.” Related to BDI items 4 and 15.
4. Feeling depressed:
  Experiencing sadness or depression, for example, “The patient has been feeling sad since their relative passed away.” Related to BDI items 1 (“I am so sad and unhappy that I can’t stand it.”) and 2 (“I feel the future is hopeless and that things cannot improve.”).
5. Feeling anxious:
  Feeling anxious, overwhelmed, or stressed, for example, “The patient is reporting elevated levels of stress caused by recent personal events.” Related to BDI item 20 (“I am so worried about my physical problems that I cannot think of anything else.”).
6. Feeling down:
  Experiencing low mood, for example, “The patient’s mood has recently lowered.” Related to BDI items 1 and 2.
7. Irritability:
  Anger or frustration directed at guardians or clinical staff, for example, “The patient seemed very annoyed by our questions.” Related to BDI item 11 (“I feel irritated all the time.”).
8. Mental health concerns:
  General or unspecified mental health concerns, for example, “Patient’s school counselor noted concerns regarding the patient’s mental health state.” Not directly related to a BDI item but associated with PHQ-2 (“Feeling down, depressed, or hopeless?”).
9. Sleep problems:
  Issues with sleep, for example, “The patient complains about having difficulty falling asleep at night and waking up in the morning.” Related to BDI items 16 (“I wake up several hours earlier than I used to and cannot get back to sleep.”) and 17 (“I am too tired to do anything.”).
10. High appetite:
  Increased appetite or overeating, for example, “The patient has gained 10+ pounds since their visit two months ago.” Not directly related to a BDI item, but associated with PHQ-5 (“Poor appetite or overeating?”).
11. Low appetite:
  Reduced appetite or weight loss, for example, “Patient’s guardian reports that it is a daily struggle to ensure that the patient consumes their meals.” Related to BDI items 18 (“I have no appetite at all anymore.”) and 19 (“I have lost more than fifteen pounds.”).
12. Weight change:
  Uncontrolled weight changes, for example, “One of the patient’s concerns is weight change.” Not directly related to a BDI item but associated with PHQ-5 (“Poor appetite or overeating?”).
13. Little energy:
  Persistent fatigue or tiredness without a clear cause, for example, “Patient’s guardian reports that the patient is always complaining about feeling tired.” Related to BDI item 17 (“I am too tired to do anything.”).
14. Self-loathing:
  Negative self-image or self-worth issues, for example, “The patient mentioned feeling worthless from time to time.” Related to BDI items 7 (“I hate myself.”) and 14 (“I believe that I look ugly.”).
15. Abnormal behavior:
  Disoriented behavior or unusual speech patterns, for example, “The patient looked disoriented at times, and their movements were erratic.” Not directly related to a BDI item but associated with PHQ-8 (“Moving or speaking so slowly that other people could have noticed? Or being so fidgety or restless that you have been moving around a lot more than usual?”).
16. Suicidal thoughts:
  Suicidal ideation, for example, “The patient has been referred to counseling due to suicidal tendencies.” Related to BDI item 9 (“I would kill myself if I had the chance.”).

### Observations on Annotation Patterns

Notably, “feeling anxious” was the most frequently annotated symptom, appearing in 14 notes, followed by “feeling depressed” in 13 notes (see Table 1). In contrast, symptoms like “irritability,” “self-loathing,” and “neglecting activities” were less frequently observed, appearing in only 2 notes each.

Symptoms tied to sadness and anxiety, particularly those in Q2, were commonly documented because both patients and their guardians tended to disclose these feelings more readily. Progress notes often included complaints related to school performance, peer relationships, or family stress. Furthermore, the frequent co-occurrence of anxiety and depression increased the likelihood of these symptoms being recorded.

Nurse notes emerged as particularly valuable sources of information, as they often captured nuanced behaviors and sensitive disclosures that might not have been documented in formal physician notes. For example, nurse notes frequently included observations about behaviors such as reluctance to engage in activities or fear of parental judgment, which were otherwise difficult to elicit.

Conversely, symptoms requiring introspection, such as guilt or self-loathing (captured in Q6), were documented less frequently. These symptoms are inherently harder to observe, requiring proactive elicitation by clinicians. Adolescent patients, in particular, may lack the vocabulary or emotional insight to express such feelings, making these symptoms less likely to be noted unless specifically probed during consultations. Additionally, clinicians may focus more on quantifiable values, such as weight fluctuations and missed school days, rather than delving into more abstract or internalized symptoms.

Behavior-based symptoms, such as “neglecting activities,” were described in highly variable contexts, making consistent annotation challenging. For example, some notes stated, “the patient quit drama club,” while others noted, “the guardian needs to encourage the patient to do drama,” or even “the patient has not been skipping drama class very often.” This variability necessitated careful interpretation by annotators to ensure accurate symptom categorization.

Lastly, prominent symptoms were sometimes recorded in an ambiguous manner. Terms like “weight concerns” or “mental health concerns” were frequently mentioned without specifying the exact nature of the problem. This ambiguity underscores the complexity of accurately detecting and categorizing symptoms in clinical notes.

All these observations emphasize the challenge of detecting depression symptoms in the adolescent setting.

**Table 1:** Summary of annotated positive symptom mentions mapped to PHQ-9a questions, based on manual annotations of 85 clinical notes from 22 patients, ages 15-17 years. We highlight symptom semantics (eg, sadness and loss of interest) and whether observed changes were increased (↑), decreased (↓), or fluctuating (↑↓) relative to each patient’s baseline.

Question	PHQ	Semantics	Notes, n	Average words, n
Not going to school^b^	Q1	↓ Interest	3	14
Neglecting activities	Q1	↓ Interest	2	15
No motivation	Q1	↓ Interest	9	19
Feeling depressed	Q2	Sadness	13	14
Feeling anxious	Q2	Sadness	14	13
Feeling down	Q2	Sadness	3	13
Irritability	Q2	Sadness	2	12
MH^c^ concerns	Q2	Sadness	5	19
Sleep problems	Q3	↓↑ Sleep	7	16
High appetite	Q4	↑ Appetite	5	24
Low appetite	Q4	↓ Appetite	7	16
Weight change	Q4	↓↑ Appetite	8	20
Little energy	Q5	↓ Energy	3	14
Self-loathing	Q6	↓ Self-image	2	12
Abnormal behavior	Q8	↓↑ Activity	4	19
Suicidal thoughts	Q9	Suicidality	4	19

^a^PHQ-9: 9-item Patient Health Questionnaire.

^b^For instance, “not going to school” is linked to loss of interest (PHQ Q1) with 3 notes averaging 14 words each.

^c^MH: mental health.

### LLM Detection Quality

We assess the quality of LLM detection by calculating *F*_1_-score, precision, and recall at the note level for the positive class. Precision quantifies the proportion of sentences identified as relevant by the model (positive predictions) that are actually relevant. Recall indicates the proportion of relevant sentences in the dataset that were successfully identified by the model. The *F*_1_-score is a metric that combines precision and recall into a single measure, representing the harmonic mean of the two, and is used to evaluate a model’s balance between false positives and false negatives.

We report results for 85 notes from 22 patients (see Symptom Annotation Process in Methods) in 3 tables (see Multimedia Appendices 2-4) for the 3 metrics, respectively.

Our general observation suggests that all the LLMs outperform the naive word-match baseline (0.55 average *F*_1_-score over LLMs vs 0.22 average *F*_1_-score of our baseline). FLAN-T5 achieves the highest average *F*_1_-score (0.65), followed closely by Phi (0.51). Llama 3-70B scores lowest overall (0.48). FLAN-T5 demonstrates consistent performance across most categories, with high scores in “sleep problems” (0.92) and “self-loathing” (0.80), showing its strength in precision. However, it performs moderately in some categories such as “feeling depressed” (0.55), “feeling anxious” (0.50), and “weight change” (0.47). Phi performs competitively, excelling in “feeling depressed” (0.69), “feeling anxious” (0.74), and “weight change” (0.78), indicating tendencies in balanced precision/recall. Llama also shows particular strength in more general-domain categories like “feeling anxious” (0.76) and “high appetite” (0.67), but its performance varies more significantly across other categories, such as very low scores in “irritability” (0.22) and “feeling down” (0.13).

Confirming our previous observations, FLAN-T5 consistently achieves the highest average precision (0.78), outperforming Phi (0.44) and Llama 3 (0.33). This suggests FLAN-T5 is better for settings where false positives are not desirable due to time constraints.

Llama 3 leads in recall with an average of 0.90, significantly higher than Phi (0.60) and FLAN-T5 (0.55). Llama’s high recall can be attributed to its tendency to overgeneralize when depressive symptoms are present, assuming that all symptoms are applicable across various depression-related queries. For example, if a general feeling of sadness is mentioned in the notes, Llama may incorrectly infer that the patient is overeating due to the correlation between the two symptoms. In addition, in one of the notes, the mention of anxiety led Llama to infer irritability. Additionally, we observed cases where Llama produced false positives based on remote premises. A brief administrative message about laboratory test results getting delayed was interpreted by Llama as an indication that the patient was feeling sad. The categories most affected by this tendency were *mental health concerns* and *feeling down*, with relatively high recall of 1 and 0.67, but precision of only 0.15 and 0.07. Phi ranks as the second-best model for both precision and recall.

Regarding symptom-specific insights, symptoms like “not going to school,” “neglecting activities,” “no motivation,” “feeling depressed,” “irritability,” “sleep problems,” “high appetite,” and “abnormal behavior” are detected with perfect precision by FLAN-T5, reflecting more distinct presentations of these symptoms. FLAN-T5 gives the lowest precision for “MH concerns” (0.25) and “feeling down” (0.40). Those categories often involve broader language, encompassing a wide range of mental health–related observations that may not directly correspond to depressive symptoms. For example, broad references to “mental health issues” in a note containing a past diagnosis of bipolar disorder or attention-deficit/hyperactivity disorder (ADHD) could be discarded by the model in relation to the context.

“Not going to school,” “neglecting activities,” “feeling anxious,” “MH concerns,” “sleep problems,” “low appetite,” “little energy,” “self-loathing,” and “suicidal thoughts” show the perfect recall for Llama 3. This is likely due to the model’s tendency to broadly associate any general depressive language with these categories. For example, phrases like “struggling with everyday tasks” or “feeling overwhelmed” might trigger multiple associations, leading to higher recall at the expense of precision.

“Feeling down” (0.67), “irritability” (0.50), and “abnormal behavior” (0.75) show the lowest Llama 3 recall. This lower recall can be attributed to several factors. “Feeling down” is, on the one hand, often expressed in less direct or subtle terms compared to other symptoms. “Irritability” is a symptom that can overlap with other emotional states, such as frustration or anger, and hence not picked up by the model. “Abnormal behavior” appears primarily in highly specific contexts, such as detailed behavioral observations by clinicians, and can be mistaken for some other external factors.

In spite of the successes, we noticed that LLMs faced challenges in analyzing clinical notes due to their complex structure, where mixed content such as summarized complaints, clinician advice, and historical visit details can obscure relevant context. Another challenge stems from sections listing test results, particularly when only the final PHQ-9 score is presented. When the score is elevated, models tend to overgeneralize, incorrectly assuming that all depression-related symptoms apply to the patient.

In conclusion, in our data, FLAN-T5 demonstrates good precision, making it effective for accurate and targeted symptom identification in time-sensitive screenings, while Phi’s higher recall relative to FLAN-T5 makes it more suited for comprehensive screening tasks.

### Utility of LLM Extracts for Depression Screening

To understand how well LLM-derived symptom annotations capture clinically meaningful signals for differential diagnosis, we applied our Flan-based symptom detector to the note corpus associated with the 15-17-year-old cohort (924 patients) from our case-control classification experiment (see Utility of LLM Detection for Depression Screening in Methods). We selected Flan for this analysis because it demonstrated the strongest balance of precision and recall. This makes this model better aligned with the practical constraints of real-world screening, enabling more accurate symptom identification without overtriggering.

Table 2 illustrates the number of clinical notes associated with each symptom detected by FLAN-T5. The most frequently detected symptoms were mental health (MH) concerns (433 notes), followed by feeling down (411 notes) and sleep problems (396 notes). The overrepresentation of certain symptoms, such as sleep-related issues, may result from the mandatory documentation of these topics in clinical notes even in the absence of any particular issues. These results suggest that general mental health issues and mood-related concerns are better documented in clinical notes than we have seen in our initial annotation sample. Conversely, symptoms like high appetite (57 notes), abnormal behavior (88 notes), and no motivation (68 notes) were least frequently detected. The table highlights significant differences in the prevalence of symptoms between cases and controls, with an overall average of 72% (2605/3595) of symptoms documented in cases and 28% (990/3595) in controls. Symptoms such as feeling depressed (240/280, 86%), feeling down (341/411, 83%), self-loathing (206/234, 88%), and suicidal thoughts (237/279, 85%) are mainly concentrated in case notes confirming mental health issues. Symptoms like sleep problems (case: 261/396, 66%; control: 135/396, 34%) and MH concerns (case: 272/433, 63%; control: 161/433, 37%) show a more balanced distribution due to the documentation practices mentioned above.

**Table 2:** Number of notes per symptom detected by FLAN-T5 for the differential diagnosis use case (15-17-year-old cohort of 924 patients).

Question	All, n	Case, n (%)	Control, n (%)
Not going to school	91	53 (58)	38 (42)
Neglecting activities	210	126 (60)	84 (40)
No motivation	68	54 (79)	14 (21)
Feeling depressed	280	240 (86)	40 (14)
Feeling anxious	271	211 (78)	60 (22)
Feeling down	411	341 (83)	70 (17)
Irritability	109	73 (67)	36 (33)
MH^a^ concerns	433	272 (63)	161 (37)
Sleep problems	396	261 (66)	135 (34)
High appetite	57	42 (74)	15 (26)
Low appetite	291	198 (68)	93 (32)
Weight change	106	64 (60)	42 (40)
Little energy	271	170 (63)	101 (37)
Self-loathing	234	206 (88)	28 (12)
Abnormal behavior	88	57 (65)	31 (35)
Suicidal thoughts	279	237 (85)	42 (15)
All positive notes/average	3595	2605 (72)	990 (28)

^a^MH: mental health.

Overall, we observed very good quality of the extracts provided by Flan. For instance, the system identified “not going to school” as being linked to 25 absences due to chronic illness episodes. Similarly, symptoms like low appetite were flagged describing urgent referrals to dietitians and psychologists: “Patient’s guardian is highly concerned with their weight and eating habits. They consulted a dietitian who urgently referred them to a psychologist. The patient denies feeling anorexic or having body image problems” (paraphrased). Lastly, extracted evidence relating to suicidal thoughts also provided some good insights as well: “Patient’s guardian called stating that the patient is having thoughts about self-harm. They believe that the patient is safe with them at the moment but requires an urgent appointment.”

The observed errors in symptom detection by FLAN-T5 often align with misinterpretations due to figurative language. For example, when a note mentioned that a patient had a “good appetite” as part of their recovery from an illness, FLAN-T5 incorrectly detected overeating.

Table 3 presents the classification performance of the evaluated machine learning models in distinguishing between case and control depression groups. Among the models tested, the SVM achieved the highest overall performance, with an AUC-ROC of 0.71 (95% CI 0.70-0.72) and an *F*_1_-score of 0.62, whereas logistic regression achieved the highest positive-class precision (0.792 compared with 0.78 for the SVM). Across the evaluated classifiers, the mean AUC-ROC was 0.68, representing an approximately 8-point improvement over the BigBird transformer baseline (AUC-ROC 0.60), which was trained directly on the most recent raw clinical note without symptom-level feature extraction. Although the BigBird baseline achieved a recall comparable to the machine learning models, its lower precision (0.43) indicates a tendency to overpredict depression cases despite the balanced evaluation dataset. These findings confirm the utility and discriminative power of our LLM-based feature extraction procedure.

**Table 3:** Performance of machine learning models for depression diagnosis classification in the differential diagnosis use case (15-17-year-old cohort; n=924). Results are reported as mean AUC-ROC^a^ with 95% CI obtained from repeated stratified 5-fold cross-validation (20 repeats). Statistical significance was assessed by paired bootstrap comparison of AUC-ROC against logistic regression (2000 bootstrap samples). Full confidence intervals for F1-score, precision, recall, and additional statistical comparisons are provided in Multimedia Appendix 5.

Model	AUC-ROC (95% CI)	*F*_1_-score	Precision	Recall
Logistic regression (LR)	0.682 (0.674-0.690)	0.591	0.792	0.471
Decision tree (DT)^b^	0.615 (0.607-0.624)	0.571	0.699	0.483
Random forest (RF)	0.680 (0.672-0.688)	0.613	0.709	0.540
Support vector machine (SVM)^c^	0.707 (0.700-0.715)	0.621	0.782	0.515
Multilayer perceptron (MLP)^c^	0.693 (0.685-0.700)	0.617	0.742	0.528
BigBird transformer baseline	0.600	0.483	0.430	0.550

^a^AUC-ROC: area under the receiver operating characteristic curve.

^b^Significantly lower than logistic regression.

^c^Significantly higher than logistic regression (95% CI for ∆AUC excludes 0).

## Discussion

### Statement of the Key Findings

This study explored the potential of using LLMs to identify depressive symptoms within adolescent EHRs. By evaluating the off-the-shelf performance of 3 state-of-the-art LLMs (FLAN-T5, Phi, and Llama 3-70B) on detecting PHQ-9-related symptoms in free-form text, we demonstrate the potential of these models to improve the consistency of depression screening in the challenging adolescent context.

This study should be interpreted as a proof-of-concept evaluation rather than a benchmark of language model architectures. Although the models were evaluated on the same annotation task, they differed in prompting strategy (zero-shot versus few-shot), task formulation (natural language inference versus question answering), and context processing due to model-specific input constraints. Consequently, the observed performance differences likely reflect both architectural characteristics and experimental design choices.

Our key findings showed that LLMs, particularly the smaller FLAN-T5 model, achieved 100% precision in several of the 16 symptom categories. These results suggest that LLMs can support mental health screening by providing accurate symptom detection, which can be useful for streamlining the diagnostic process. Within the prompting configurations evaluated in this pilot study, FLAN-T5 demonstrated the best overall balance between precision and recall. In high-stakes settings where sensitivity is critical and alert fatigue is less important, Llama 3 may be the preferred option due to its substantially higher recall (0.90), despite very low precision.

Notably, the simple word-search baseline achieved higher precision than Phi and Llama 3. This reflects the conservative keyword-matching strategy, which generates fewer false positives but misses many true symptom mentions, leading to substantially lower recall and overall *F*_1_-score than the LLM-based approaches.

### How This Study Adds/Supports Previous Studies in This Area?

This study builds on prior research by applying LLMs to annotate depressive symptoms directly from clinical notes [24-27].

This study supports the utility of off-the-shelf LLMs like FLAN-T5 for detecting symptom-related evidence [16,17], a method previously underexplored in pediatric mental health contexts. Our findings suggest that smaller models may provide competitive performance in this information extraction task when used in appropriate prompting configurations. However, this observation should be interpreted cautiously because the evaluated models differed not only in architecture but also in task formulation, prompting strategy, and context handling [35].

The efficacy and safety of such tools in real-world clinical settings remain to be thoroughly investigated [36]. This study highlights the advantage of using real-world data to assess these tools, particularly when applied to depression. While the majority of studies suggest that LLMs cannot replace human therapists [37,38], they can serve as supplementary tools. These tools can integrate human clinical insights to improve therapeutic processes.

However, challenges remain, including issues related to data bias and the necessity of human oversight. These challenges emphasize the need for careful integration of LLMs into existing health care frameworks [39,40]. It is crucial to augment rather than replace traditional diagnostic and treatment practices to ensure the effective and safe use of these tools in clinical settings, especially for treating depression. We have proposed an LLM-based approach to detect PHQ-9 symptoms from unstructured data, such as doctor observations and patient replies, to support traditional practices.

### Strengths and Limitations

The strengths of this work lie in its novel application of LLMs for text-based evidence detection in a domain where annotated data is particularly sparse and texts are very heterogeneous (mental health clinical notes). We also use the LLM extracts as features to train a machine learning–based screening system as a proxy to human screening, which outperforms a strong baseline not using our feature extracts.

By leveraging the generalization capabilities of LLMs in an off-the-shelf setting, this approach avoids the need for costly and time-consuming manual annotation of training datasets. Furthermore, the ethical framework used ensures that the models are used solely for evidence detection, maintaining clinician agency and control over the diagnostic process. In addition, the best-precision model, FLAN-T5, being a relatively small and computationally efficient model, is well-suited for deployment in clinical settings where resources are limited.

The study has some limitations. First, the evaluation relies on a small annotated dataset (85 notes from 22 patients), which restricts the robustness of our accuracy estimates. Second, our results are constrained to an adolescent subset (ages 15-17 years) drawn from a broader dataset spanning ages 6-24 years. Third, more extensive experimentation is required to support comparisons between the models statistically. Additionally, differences in prompting strategy (few-shot for Phi and Llama versus zero-shot for FLAN-T5), task formulation (question answering versus natural language inference), and context processing may confound architectural differences with prompting effects. Fourth, while our prompting framework eliminates the need for annotated training data, it may lead to variations in performance across different clinical settings or note structures. Importantly, a proper multiannotator validation process with human experts is necessary to fully support the reliability of the ground-truth labels. Finally, the study focuses on extracting PHQ-9-relevant information, which, while validated, does not encompass the full spectrum of depressive symptoms or contextual factors.

### Implications

The implications of this study are significant for the field of mental health diagnostics, particularly in adolescent care. By addressing the challenges of data scarcity and heterogeneity, LLM-based tools can enhance the efficiency and consistency of depression screening, particularly in resource-limited settings. Moreover, the interpretability of extracted text segments allows for greater transparency and facilitates clinicians’ trust in AI-assisted tools. This approach also provides a foundation for future research into using LLMs for feature extraction for downstream AI across mental health conditions.

### Future Work

Clinical adoption of LLM-assisted diagnostics will require addressing several challenges. Models must be validated across diverse patient groups and clinical contexts to ensure reliability. Safeguards against overgeneralization and hallucination are essential to maintain trust. Workflow integration requires taking into account alert fatigue and ensuring that model outcomes are transparent and interpretable. Furthermore, practical considerations, including data security, governance, and model size, will be critical for safe and sustainable deployment. Further research should focus on refining these models, addressing their limitations, and validating their performance in real-world clinical settings.

### Conclusions

The results demonstrate the feasibility of using off-the-shelf LLMs for symptom detection in adolescent clinical notes. Within the evaluated configurations, FLAN-T5 provided the most favorable balance between precision and recall, with an average precision of 0.78.

Furthermore, LLM-based annotation features substantially improved the performance of machine learning models for case-control classification, demonstrating their added value for clinical decision support.

While challenges remain in ensuring reliability and generalizability, the findings underscore the promise of integrating LLMs into clinical workflows to enhance the detection and management of depression in young patients. More specifically, relatively lightweight LLMs such as FLAN-T5 could be integrated as supplementary tools that automatically surface symptom indications from extensive patient history documentation. This mechanism would improve the consistency of depression screening as well as increase in-person time. In adolescent primary care, this could help compensate for incomplete and inconsistent PHQ-9 data.

## Appendix

Multimedia Appendix 1 PHQ cohort characteristics. PHQ: Patient Health Questionnaire.

Multimedia Appendix 2 Symptom mention detection positive precision results.

Multimedia Appendix 3 Symptom mention detection positive recall results.

Multimedia Appendix 4 Symptom mention detection F1-score results.

Multimedia Appendix 5 Bootstrap estimates of classifier performance from repeated stratified 5-fold cross-validation (20 repeats).

## Abbreviations

ADHD: attention-deficit/hyperactivity disorder
AUC-ROC: area under the receiver operating characteristic curve
BDI: Beck Depression Inventory
BERT: Bidirectional Encoder Representations from Transformers
CCHMC: Cincinnati Children’s Hospital Medical Center
CDC: Centers for Disease Control and Prevention
DSM-IV: Diagnostic and Statistical Manual of Mental Disorders, Fourth Edition
EHR: electronic health record
FLAN-T5: Finetuned Language Net, Text-to-Text Transfer Transformer
ICD: International Statistical Classification of Diseases and Related Health Problems
LDA: latent Dirichlet allocation
Llama: Large Language Model Meta AI
LLM: large language model
MH: mental health
MLP: multilayer perceptron
NLP: natural language processing
PHQ: Patient Health Questionnaire
SVM: support vector machine

## Data Availability

The data are derived from electronic health records (EHRs) at Cincinnati Children’s Hospital Medical Center (CCHMC). These data are not publicly available. However, deidentified data that support the findings of this study can be made available through collaboration. This is subject to institutional approvals and compliance with applicable data protection regulations. The authors affirm that all necessary permissions for data use were obtained prior to conducting the study.
