# Supplementary material for: Assisting Screening of Adolescent Depression With Large Language Models as Symptom Detectors: Algorithm Development and Validation
Source: JMIR Form Res. 2026 Oct 21;10:e71614. doi: 10.2196/71614 (PMC13735180; doi:10.2196/71614)
Supplement: Supplementary file 1 [file formative_v10i1e71614_app1.pdf]

Multimedia Appendix 1: PHQ Cohort Characteristics (Age Groups with Sufficient Data)

Table S1: Summary statistics for extracted PHQ data.

Age Bin	N patients in cohort	% with at least one PHQ	% with at least one PHQ-9	% with at least one PHQ-2	% with at least two PHQ	% with at least two PHQ-9	% with at least two PHQ-2	N visits with PHQ taken	N patients with complete PHQ record
6	94	**100%**	7%	**91%**	23%	0%	22%	13	3
12	2340	98%	17%	83%	45%	0%	**45%**	642	507
13	3323	99%	46%	65%	35%	17%	11%	937	1076
14	4015	98%	52%	56%	36%	17%	8%	**973**	1274
15	**4448**	98%	56%	53%	36%	20%	11%	931	1491
16	4296	98%	70%	46%	52%	**35%**	12%	783	**1565**
17	3463	96%	70%	48%	55%	**35%**	15%	596	1452
18	1201	98%	76%	51%	60%	**35%**	16%	285	1010
19	677	98%	**81%**	49%	**61%**	**35%**	22%	165	685
20	454	99%	78%	46%	59%	30%	22%	119	465
21	255	**100%**	69%	55%	50%	26%	15%	62	193
22	147	**100%**	52%	63%	30%	11%	11%	27	83
23	107	**100%**	68%	31%	8%	0%	7%	13	33
24	70	**100%**	61%	37%	12%	11%	0%	8	19
Av.	1778	99%	57%	55%	40%	19%	16%	397	704

N denotes the number of patients or visits. “% with at least one/two PHQ” represents the proportion of patients with at least one recorded PHQ score. Approximately 2% of cases include only the binary indicator, so this percentage may not equal 100%. “% with at least one/two PHQ-9” and “% with at least one/two PHQ-2” represent the proportions of cohort patients with at least one recorded PHQ score for the corresponding questionnaire; a recorded score does not necessarily indicate that the complete questionnaire was available. “N visits with PHQ taken” denotes the number of visits with a recorded PHQ-administration indicator. “N patients with complete PHQ record” denotes patients in the source PHQ database who completed at least one full item-level PHQ-9. This population was identified before the study inclusion and exclusion criteria were applied and may therefore include patients who were not part of the final study cohort. The complete source cohort comprised 28,172 patients aged 6–24 years. The age-specific results presented in this table are restricted to age groups for which sufficient PHQ data were available.
