# Supplementary material for: Assisting Screening of Adolescent Depression With Large Language Models as Symptom Detectors: Algorithm Development and Validation
Source: JMIR Form Res. 2026 Oct 21;10:e71614. doi: 10.2196/71614 (PMC13735180; doi:10.2196/71614)
Supplement: Supplementary file 2 [file formative_v10i1e71614_app2.pdf]

Multimedia Appendix 2: Symptom Mention Detection Positive Precision Results

Table S1: Positive Precision of Symptom Mention Detection over the In-House Test Set.

Category	Flan-T5	Phi	Llama 3 70B	Baseline
Not going to school	1.00	0.50	0.38	1.00
Neglecting activities	1.00	0.50	0.20	0.00
No motivation	1.00	0.25	0.26	N/A
Feeling depressed	1.00	0.62	0.50	0.80
Feeling anxious	0.83	0.77	0.61	N/A
Feeling down	0.40	0.12	0.07	0.09
Irritability	1.00	0.50	0.14	N/A
MH concerns	0.25	0.13	0.15	N/A
Sleep problems	1.00	0.80	0.33	0.25
High appetite	1.00	N/A	0.57	N/A
Low appetite	0.71	0.60	0.54	N/A
Weight change	0.60	0.70	0.24	0.50
Little energy	0.43	0.29	0.27	1.00
Self-loathing	0.67	0.33	0.33	N/A
Abnormal behavior	1.00	0.00	0.33	N/A
Suicidal thoughts	0.60	0.50	0.31	N/A
Average	0.78	0.44	0.33	0.52

N/A stands for zero division.
