# Supplementary material for: Assisting Screening of Adolescent Depression With Large Language Models as Symptom Detectors: Algorithm Development and Validation
Source: JMIR Form Res. 2026 Oct 21;10:e71614. doi: 10.2196/71614 (PMC13735180; doi:10.2196/71614)
Supplement: Supplementary file 3 [file formative_v10i1e71614_app3.pdf]

Multimedia Appendix 3: Symptom Mention Detection Positive Recall Results

Table S1: Positive Recall of Symptom Mention Detection over the In-House Test Set.

Category	Flan-T5	Phi	Llama 3 70B	Baseline
Not going to school	0.33	0.67	1.00	0.33
Neglecting activities	0.50	1.00	1.00	0.00
No motivation	0.22	0.67	0.89	0.00
Feeling depressed	0.38	0.77	0.92	0.31
Feeling anxious	0.36	0.71	1.00	0.00
Feeling down	0.67	0.67	0.67	0.33
Irritability	0.50	0.50	0.50	0.00
MH concerns	0.40	0.60	1.00	0.00
Sleep problems	0.86	0.57	1.00	0.29
High appetite	0.20	0.00	0.80	0.00
Low appetite	0.71	0.86	1.00	0.00
Weight change	0.38	0.88	0.88	0.25
Little energy	1.00	0.67	1.00	0.67
Self-loathing	1.00	0.50	1.00	0.00
Abnormal behavior	0.50	0.00	0.75	0.00
Suicidal thoughts	0.75	0.50	1.00	0.00
Average	0.55	0.60	0.90	0.14
