# Supplementary material for: Assisting Screening of Adolescent Depression With Large Language Models as Symptom Detectors: Algorithm Development and Validation
Source: JMIR Form Res. 2026 Oct 21;10:e71614. doi: 10.2196/71614 (PMC13735180; doi:10.2196/71614)
Supplement: Supplementary file 4 [file formative_v10i1e71614_app4.pdf]

Multimedia Appendix 4: Symptom Mention Detection F1 Results

Table S1: Positive F1 of Symptom Mention Detection over the In-House Test Set. Average F1 is computed from averaged precision/recall rather than averaged per-category F1 values.

Category	Flan-T5	Phi	Llama 3 70B	Baseline
Not going to school	0.50	0.57	0.55	0.50
Neglecting activities	0.67	0.67	0.33	N/A
No motivation	0.36	0.36	0.40	N/A
Feeling depressed	0.55	0.69	0.65	0.45
Feeling anxious	0.50	0.74	0.76	N/A
Feeling down	0.50	0.20	0.13	0.14
Irritability	0.67	0.5	0.22	N/A
MH concerns	0.31	0.21	0.26	N/A
Sleep problems	0.92	0.67	0.50	0.27
High appetite	0.33	N/A	0.67	N/A
Low appetite	0.71	0.71	0.70	N/A
Weight change	0.47	0.78	0.38	0.33
Little energy	0.60	0.40	0.43	0.80
Self-loathing	0.80	0.40	0.50	N/A
Abnormal behavior	0.67	N/A	0.46	N/A
Suicidal thoughts	0.67	0.50	0.47	N/A
Average	0.65	0.51	0.48	0.22

N/A stands for zero division.
