# Supplementary material for: Assisting Screening of Adolescent Depression With Large Language Models as Symptom Detectors: Algorithm Development and Validation
Source: JMIR Form Res. 2026 Oct 21;10:e71614. doi: 10.2196/71614 (PMC13735180; doi:10.2196/71614)
Supplement: Supplementary file 5 [file formative_v10i1e71614_app5.pdf]

Multimedia Appendix 5: Classification Performance

Table S1: Bootstrap estimates of classifier performance from repeated stratified 5-fold cross-validation (20 repeats). Values are reported as mean with 95% confidence intervals (CI). Logistic Regression (LR) served as the reference model. Statistical significance was assessed using paired bootstrap comparison (2,000 bootstrap samples). * Significantly higher than LR; † significantly lower than LR (95% CI for the paired bootstrap difference excludes 0).

**Metric**	**Statistic**	**LR**	**DT**	**RF**	**MLP**	**SVM**
AUC-ROC	Value (95% CI)	0.682<br>[0.674, 0.690]	0.615†<br>[0.607, 0.624]	0.680<br>[0.672, 0.688]	0.693*<br>[0.685, 0.700]	0.707*<br>[0.700, 0.715]
F1	Value (95% CI)	0.591<br>[0.582, 0.600]	0.571†<br>[0.562, 0.580]	0.613*<br>[0.604, 0.622]	0.617*<br>[0.608, 0.625]	0.621*<br>[0.612, 0.630]
Precision	Value (95% CI)	0.792<br>[0.782, 0.803]	0.699†<br>[0.688, 0.710]	0.709†<br>[0.698, 0.720]	0.742†<br>[0.732, 0.752]	0.782†<br>[0.771, 0.792]
Recall	Value (95% CI)	0.471<br>[0.460, 0.481]	0.483*<br>[0.473, 0.493]	0.540*<br>[0.530, 0.550]	0.528*<br>[0.517, 0.538]	0.515*<br>[0.504, 0.525]
